# Identification and Functional Analysis of the *erh1* ^+^ Gene Encoding Enhancer of Rudimentary Homolog from the Fission Yeast *Schizosaccharomyces pombe*

**DOI:** 10.1371/journal.pone.0049059

**Published:** 2012-11-07

**Authors:** Marek K. Krzyzanowski, Ewa Kozlowska, Piotr Kozlowski

**Affiliations:** 1 Department of Molecular Biology, Faculty of Biology, University of Warsaw, Warsaw, Poland; 2 Department of Immunology, Faculty of Biology, University of Warsaw, Warsaw, Poland; University of Cambridge, United Kingdom

## Abstract

The *ERH* gene encodes a highly conserved small nuclear protein with a unique amino acid sequence and three-dimensional structure but unknown function. The gene is present in animals, plants, and protists but to date has only been found in few fungi. Here we report that *ERH* homologs are also present in all four species from the genus *Schizosaccharomyces*, *S. pombe*, *S. octosporus*, *S. cryophilus*, and *S. japonicus*, which, however, are an exception in this respect among Ascomycota and Basidiomycota. The ERH protein sequence is moderately conserved within the genus (58% identity between *S. pombe* and *S.* *japonicus*), but the intron-rich genes have almost identical intron-exon organizations in all four species. In *S. pombe*, *erh1^+^* is expressed at a roughly constant level during vegetative growth and adaptation to unfavorable conditions such as nutrient limitation and hyperosmotic stress caused by sorbitol. Erh1p localizes preferentially to the nucleus with the exception of the nucleolus, but is also present in the cytoplasm. Cells lacking *erh1^+^* have an aberrant cell morphology and a comma-like shape when cultured to the stationary phase, and exhibit a delayed recovery from this phase followed by slower growth. Loss of *erh1^+^* in an auxotrophic background results in enhanced arrest in the G1 phase following nutritional stress, and also leads to hypersensitivity to agents inducing hyperosmotic stress (sorbitol), inhibiting DNA replication (hydroxyurea), and destabilizing the plasma membrane (SDS); this hypersensitivity can be abolished by expression of *S. pombe erh1^+^* and, to a lesser extent, *S. japonicus erh1^+^* or human *ERH*. Erh1p fails to interact with the human Ciz1 and PDIP46/SKAR proteins, known molecular partners of human ERH. Our data suggest that in *Schizosaccharomyces* sp. *erh1^+^* is non-essential for normal growth and Erh1p could play a role in response to adverse environmental conditions and in cell cycle regulation.

## Introduction

The *enhancer of rudimentary homolog* (*ERH*) gene is present in genomes of most eukaryotic organisms including Animalia, Plantae and Protista with the noteworthy exception of Fungi in which it has not been described yet in such prominent model organisms as *Saccharomyces cerevisiae*, *Schizosaccharomyces pombe* or *Aspergillus nidulans* [1]–[8]. A typical *ERH* gene codes for a small, approx. 100 amino acid long polypeptide. Its sequence is not similar to any other protein known but a comparison between species belonging to different higher taxa shows that it has been conserved during evolution, especially in Vertebrata in which the human and zebrafish *Danio rerio* ERH proteins differ only by one conservative substitution [3]. Analysis of ERH does not reveal any important sequence motifs including a subcellular localization signal, however, studying human ERH we found that it is present predominantly in the nucleus [9]. The exact function of ERH is unknown but some have been proposed. The gene was originally identified in *Drosophila melanogaster* in which its mutation augmented the truncated wings phenotype resulted from the hypomorphic mutation in another gene, *rudimentary* (*r*). The *r* gene codes for the first three enzymatic activities of the *de novo* pyrimidine nucleotide biosynthetic pathway and the effect was only seen in the *r* mutant background. Hence, the novel gene was named *enhancer of rudimentary* and its protein product was proposed to be involved in the regulation of the metabolism of pyrimidines [1], [10]. Based on the *in vitro* phosphorylation of its protein by CK2 and the expression pattern of its mammalian homologs the same group also suggested that it could play a role in the cell cycle [3]. Later, following identification of the *Xenopus laevis* DCoH/PCD protein, a cell-type specific transcriptional cofactor as its molecular partner, ERH was indicated as a transcriptional repressor at least in some cells [4]. Also, we have shown that in all human cells ERH could interact with two proteins, a DNA replication factor Ciz1, and PDIP46/SKAR, a component of the exon junction complex involved in the regulation of cell growth, suggesting some functions of ERH in these fundamental processes [9], [11]–[13]. The only well characterized feature of ERH so far is its high resolution three-dimensional structure. ERH is a single domain protein which in accordance with its unique amino acid sequence, exhibits a novel α+β protein fold with the overall topology β1-3_10_-β2-α1-α2-β3-β4-α3 [5], [14]–[16].

Certainly, further studies are necessary to elucidate the role of ERH in the eukaryotic cell. In this context, the finding of its homologs in fungi, especially those intensively studied, could be very helpful in achieving this objective. Recent progress in the sequencing of various fungal genomes has revealed that some of them indeed possess an *ERH* homolog, e.g., *Mucor circinelloides* (genome.jgi-psf.org/Mucci2), *Batrachochytrium dendrobatidis* (genome.jgi-psf.org/Batde5) or *Schizosaccharomyces japonicus* (for all fission yeast genome sequences see the Internet addresses listed in Materials and Methods). None of those genes has been studied in detail yet but the identification of the *ERH* homolog in *S. japonicus* was especially intriguing, since the initial report on the genome of another fission yeast, *S. pombe*, a closely related and widely used model organism for genetic and cell cycle studies, did not reveal the presence of this gene [7]. Prompted by this finding we performed an independent examination of the *S. pombe* genome and identified a *bona fide* homolog of *ERH*. Not surprisingly, *ERH* was also missed in various ‘ome-wide analyses performed so far by the fission yeast community [17]–[20].

Therefore, to characterize for the first time a fungal homolog of the *ERH* gene, we cloned the *ERH* genes from all four species of the genus *Schizosaccharomyces* known to date, *S. pombe*, *S. octosporus*, *S. cryophilus* and *S. japonicus*, and investigated the role of ERH in *S. pombe* by studying gene expression and protein localization in wild-type cells, performing functional analysis using gene disruption mutants, and conducting cross-species experiments, also with human *ERH*.

## Results

The initial report on the *S. pombe* genome sequence by the Sanger Institute identified 4,824 protein-encoding genes of which none was a homolog of *ERH* [7]. However, the genomes of two other members of the genus *Schizosacccharomyces*, *S. japonicus* and *S. octosporus* were also sequenced as part of the Fungal Genome Initiative at the Broad Institute and their analysis revealed the presence of *ERH*-related sequences in both these species as loci SJAG_01867.4 and SOCG_03029.5, respectively [21]. When we evaluated these sequences we found that while the open reading frame (ORF) of the *S. japonicus* homolog was assembled correctly, the ORF of the *S. octosporus* gene did not seem to be complete (lacked one exon). In the light of the fact that these two species of the genus *Schizosaccharomyces* possess an *ERH* homolog we examined the genome of the third species of the genus, *S. pombe*, for the presence of an *ERH* homolog that could be overlooked during the initial automatic assembly of its ORFeome. Not surprisingly, after a BLAST search with the *S. japonicus ERH* homolog amino acid sequence as a query we identified a *bona fide ERH* homolog in the *S. pombe* genome (nucleotides 4066258–4066971 on chromosome 1). An update on the annotation of the *S. pombe* genome also revealed the presence of this gene as locus SPAC19G12.17 but initially its ORF did not seem to be assembled correctly in the *S. pombe* genome project database at the Sanger Institute either [22]. Finally, an *ERH* homolog was identified as locus SPOG_02632.3 in the genome of the fourth, recently discovered species from this genus, *S. cryophilus*, that was published jointly by the Stowers Institute and the Broad Institute [21], [23]. Thus, it became evident that all four currently known species of the genus *Schizosaccharomyces* possess an *ERH* gene.

### Cloning and Sequence Analysis of the *ERH* Genes and ERH Proteins from All Species of the Genus *Schizosaccharomyces*

**Figure 1:**
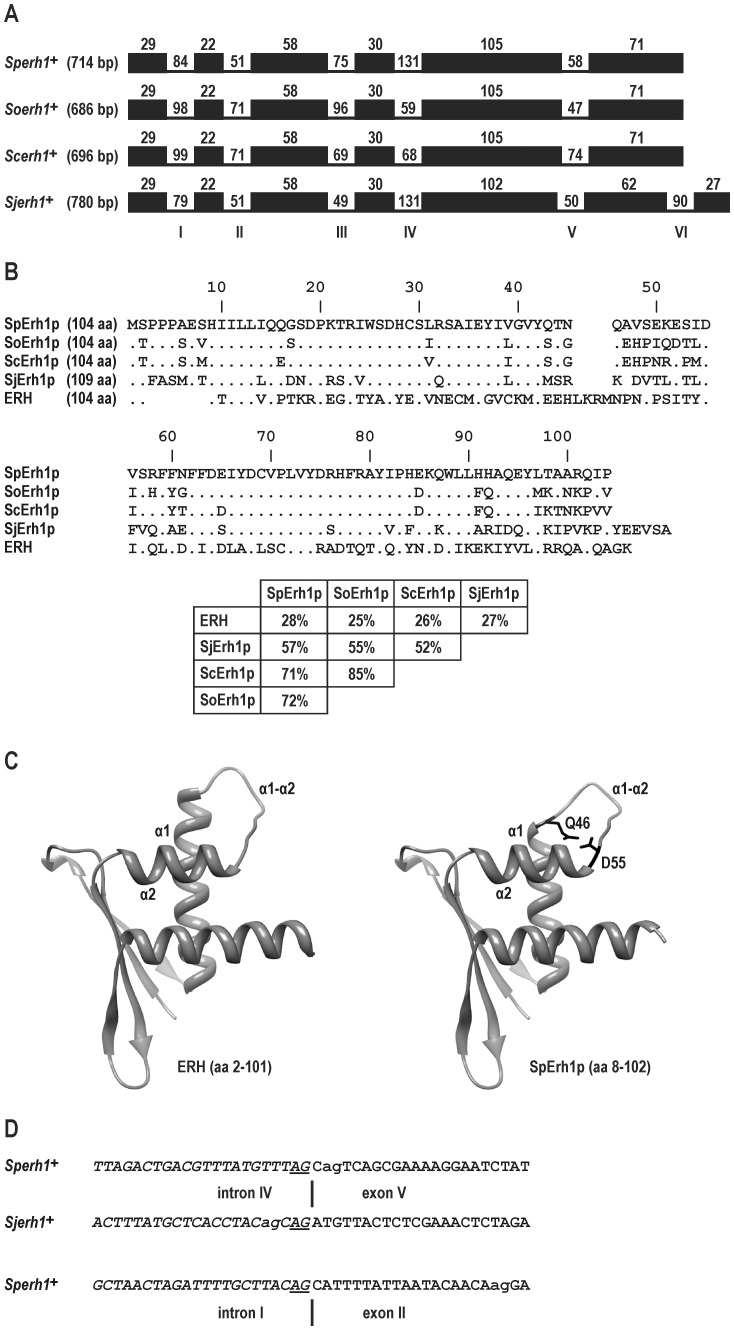
*ERH* genes and ERH proteins from four *Schizosaccharomyces* species. (A) Intron-exon organization of *ERH* genes from *S. pombe* (*Sperh1^+^*), *S. octosporus* (*Soerh1^+^*), *S. cryophilus* (*Scerh1^+^*) and *S. japonicus* (*Sjerh1^+^*). Number in parentheses gives total length of CDS plus introns. Black blocks represent exons and are drawn to scale; numbers on top give their lengths in bp. Incisions with numbers indicate intron positions and their length in bp. Consecutive introns are labeled with Roman numerals. (B) Alignment of ERH amino acid sequences from *S. pombe* (SpErh1p), *S. octosporus* (SoErh1p), *S. cryophilus* (ScErh1p) and *S. japonicus* (SjErh1p). Human ERH is shown as a reference sequence. Numbering according to SpErh1p. Number in parentheses indicates the length of the protein. Dots indicate identical residues and blanks denote missing amino acids. Table shows percent identity of sequences. (C) Predicted three-dimensional structure of Sperh1p generated by SWISS-MODEL using coordinates for human ERH from Protein Data Bank (PDB identifier: 2nmlA). Protein images produced with UCSF Chimera. Helices α1 and α2 and loop α1-α2 in both proteins and the first (Q46) and last (D55) amino acid residues of loop α1-α2 in SpErh1p are indicated. (D) Intron IV-exon V junctions in *Sperh1^+^* and *Sjerh1^+^* and intron I-exon II junction in *Sperh1^+^*. Sequences of introns are italicized. The AG sequence of the 3′ splice site is underlined and the neighboring AG is denoted by lower case. For details see text.

To characterize for the first time an *ERH* homolog from *Fungi* and to obtain correctly assembled ORFs, we cloned genomic copies and cDNA sequences of the *ERH* homologs from all four *Schizosaccharomyces* species, *S. pombe*, *S. octosporus*, *S. cryophilus* and *S. japonicus* (Figure 1A, B). All the nucleotide sequences of the cloned genomic copies were identical to those available from the Sanger Institute or the Broad Institute. The *S. pombe ERH* gene (*Sperh1^+^*) has a coding sequence (CDS) of 315 bp and codes for a protein of 104 aa (SpErh1p). It is interrupted by five short introns, so the total length from the start to the stop codon, inclusively, in the genomic sequence is 714 bp. The *S. octosporus ERH* gene (*Soerh1^+^*) has a CDS of 315 bp and codes for a 104-aa protein (SoErh1p). It is also interrupted by five introns and its total length is 686 bp. Similary, the *S. cryophilus ERH* gene (*Scerh1^+^*) CDS is 315 bp in length, codes for a 104-aa protein (ScErh1p) and is interrupted by five introns. Its total length is 696 bp. The *S. japonicus ERH* gene (*Sjerh1^+^*) CDS is 330 bp in length and codes for a 109-aa protein (SjErh1p). It is interrupted by six introns and the total length is 780 bp. All these fission yeast ERHs are similar in length to ERHs from other organisms including the 104-aa human ERH, however, the ERH domain (amino acids 3–102 in human ERH) is shortened by four (in SpErh1p, SoErh1p and ScErh1p) or five (in SjErh1p) amino acid residues, which is compensated by additional amino acid residues at the N-terminus of the protein in all four species (Figure 1B) [5], [14]–[16]. Additionally, SjErh1p has extra six amino acids at the C-terminus. The amino acid sequence of SpErh1p displays 28% identity and 54% similarity to human ERH (Figure 1B). SpErh1p has 72% and 71% identities to SoErh1p and ScErh1p, respectively, and only 57% identity to SjErh1p. Overall, only the positions of 50% of the SpErh1p amino acid residues are conserved in all four fission yeast species.

Although the SpErh1p primary structure is not highly similar to that of ERHs from other taxa and as a matter of fact the amino acid sequence of ERH is not very well preserved even within the genus *Schizosaccharomyces*, its secondary and tertiary structures seem to be highly conserved (Figure 1C). The prediction of the Sperh1p three-dimensional structure by the protein structure homology-modeling server SWISS-MODEL using the Protein Data Bank coordinates for human ERH as a template shows a single domain that has all three α helices and four β strands in the same arrangement as found in human ERH, including the long flexible loop between helices α1 and α2 (loop α1-α2, amino acids 46–55 in SpErh1p), which is the region of the weakest similarity among the four fission yeast proteins (Figure 1B) [5], [14]–[16]. The main difference concerns helix α1 which is shortened at the end by four amino acid residues (one full turn) in comparison with human ERH. The lack of another amino acid residue in SjErh1p seems to affect the length of loop α1-α2 in this protein (data not shown).

All introns have the GT sequence in the 5′ splice site and the AG sequence in the 3′ splice site. While the lengths and nucleotide sequences of the introns are rather different in the four *Schizosaccharomyces* species, their positions have been perfectly conserved during evolution (Figure 1A). *Sperh1^+^*, *Soerh1^+^* and *Scerh1^+^* have all their introns in identical positions within their CDSs, as is also true for first four introns of *Sjerh1^+^*. The fifth *Sjerh1^+^* intron is closer to the 5′ end of the ORF than the fifth introns in the other three genes because the immediately preceding fifth exon is shortened at the 5′ end by three nucleotides. This is also the reason for the aforementioned lack of one amino acid residue in the SjErh1p loop α1-α2. Interestingly, there is an AG sequence exactly three nucleotides upstream from the 3′ end of the fourth intron of *Sjerh1^+^* (Figure 1D). So it seems likely that the 3′ end of this intron “migrated” by three nucleotides into the 5′ end of the fifth exon. The last intron of *Sjerh1^+^* does not have a counterpart in any of the other genes and the interruption of the sequence coding for the poorly conserved region at the C-terminus of ERH by this intron is accompanied by the 6-amino acid extension. The introns in all four genes show a significant 5′ bias. Four out of five or six introns lie in the first half of the ERH CDS.

During cloning of the *Sperh1^+^* cDNA we also isolated two incorrectly spliced sequences (Figure 1D). One of them resulted from the excision of the fourth intron using the AG sequence lying three nucleotides downstream from its correct 3′ splice site and coded for a 103-aa polypeptide that lacked one amino acid residue (alanine) in loop α1-α2. In this respect it resembled the splicing pattern of *Sjerh1^+^*. The other cDNA resulted from the excision of the first intron together with most of the second exon due to using the AG sequence located 20 nucleotides downstream from its correct 3′ splice site and coded for a 28-aa oligopeptide only because of the reading frame shift and creation of a premature stop codon. During cloning of the *Scerh1^+^* cDNA we isolated one sequence that lacked the whole fourth exon presumably because of the co-excision of the third and fourth introns. We did not find any incorrectly spliced sequences during cloning of the cDNAs of the two other genes.

### Initial Characterization of the *erh1^+^* Gene and Erh1p Protein from *S. pombe*

**Figure 2:**
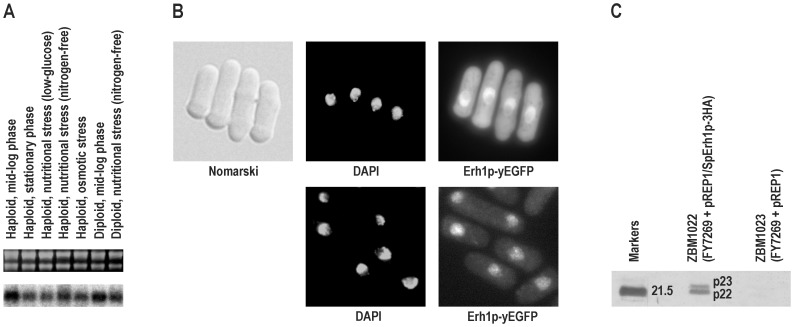
Expression of *erh1^+^* and characterization of Erh1p in *S. pombe*. (A) Northern analysis of *erh1^+^* transcript. Total RNA from haploid strain FY12697 cultured to mid-log phase in YES, cultured to stationary phase in YES, subjected to nutritional stress in low-glucose EMM2, subjected to nutritional stress in EMM2-N or subjected to hyperosmotic stress in YES supplemented with 2 M sorbitol and a diploid constructed freshly by crossing strains FY12697 and FY7519, cultured to mid-log phase in YES or subjected to nutritional stress in EMM2-N, separated by 1.2% formaldehyde/agarose gel electrophoresis and stained with ethidium bromide (upper panel) followed by transfer to membrane and hybridization with radiolabeled *erh1^+^* cDNA (lower panel). (B) Intracellular localization of Erh1p. Upper series of images, cells expressing yEGFP-tagged Erh1p from pREP1 (strain ZBM1021) visualized with Nomarski Interference Contrast, nuclei stained with DAPI and localization of yEGFP-tagged Erh1p determined by direct fluorescence; lower series of images, cells expressing yEGFP-tagged Erh1p from *erh1^+^* chromosomal locus (strain ZBM1028), nuclei stained with DAPI and localization of yEGFP-tagged Erh1p determined by direct fluorescence. (C) Identification of two forms of Erh1p. Immunoprecipitates with anti-HA monoclonal antibody from lysed cells expressing 3HA-tagged Erh1p (strain ZBM1022) or negative control (strain ZBM1023) separated by 15% SDS/polyacrylamide gel electrophoresis and stained with silver. Protein molecular mass standard of 21.5 kDa is shown to the left. Both protein bands (p22 and p23) were identified by tandem mass spectrometry as Erh1p-3HA.

To establish the expression pattern of *erh1^+^* during vegetative growth and adaptation to unfavorable conditions in *S. pombe* we isolated total RNA from the major stages and performed Northern analysis using radiolabeled *erh1^+^* cDNA as a probe (Figure 2A). The *erh1^+^* transcript was present at a similar level in all stages examined: both the haploid and diploid cultured to the mid-log phase, the haploid cultured to the stationary phase, both the haploid and diploid subjected to nutritional stress in nitrogen-free EMM2 (EMM2-N) minimal medium, and the haploid subjected to nutritional stress in low-glucose EMM2 minimal medium or to hyperosmotic stress.

Erh1p was not included in proteome-wide studies on the intracellular localization of *S. pombe* proteins [19], [20]. Our analysis of its sequence did not reveal any targeting signal. Thus, to determine the intracellular localization of Erh1p we introduced plasmid pREP1/SpErh1p-link-yEGFP coding for Erh1p tagged on the C-terminus with yEGFP (yeast-enhanced green fluorescence protein) into strain FY7269 and analyzed the resulting cells (strain ZBM1021) by fluorescence microscopy (Figure 2B). Erh1p-yEGFP was clearly present in the nucleus, with the exception of the nucleolus. However, there was also a significant amount of Erh1p-yEGFP in the cytoplasm. To exclude the possibility that the presence of Erh1p-yEGFP in the cytoplasm resulted from its overexpression (pREP1 is a high-copy number expression plasmid with a strong promoter, *nmt1*), we chromosomally tagged *erh1^+^* with yEGFP at its 3′ end in the same strain (FY7269). In the resulting cells (strain ZBM1028) the expression of Erh1p-yEGFP under the control of its own promoter was weaker but the protein was still observed in the cytoplasm (Figure 2B).

To further characterize Erh1p we introduced plasmid pREP1/SpErh1p-3HA coding for Erh1p tagged on the C-terminus with 3HA (a triple hemagglutinin epitope) of the theoretical molecular weight of 16907 Da into strain FY7269. Transformant (strain ZBM1022) cells were lysed and after immunoprecipitation with an anti-HA antibody precipitates were separated by SDS/polyacrylamide gel electrophoresis and stained with silver (Figure 2C). Two strong protein bands with electrophoretic mobilities of approx. 22 and 23 kDa were consistently detected. These bands were absent in the precipitate obtained from cells of the control strain ZBM1023 (strain FY7269 transformed with plasmid pREP1). Tandem mass spectrometry analysis of these two bands unveiled the presence of numerous tryptic peptides corresponding to Erh1p-3HA in both of them (79% and 67% of protein sequence coverage for the lower and upper band, respectively), indicating that they indeed were two forms of this protein (data not shown).

### Functional Analysis of the *erh1^+^* Gene from *S. pombe*

To investigate the role of Erh1p in *S. pombe* we disrupted *erh1^+^* through replacement of the full coding region with a kanamycin cassette by double homologous recombination in the prototrophic strain ZBM1004 and auxotrophic strains FY7269 (ade^−^ leu^−^) and FY7266 (ade^−^ leu^−^ ura^−^). The obtained disruptants (*erh1Δ* strains), ZBM1005, ZBM1020 and ZBM1030, respectively, were tested for growth defects in various conditions.

**Figure 3:**
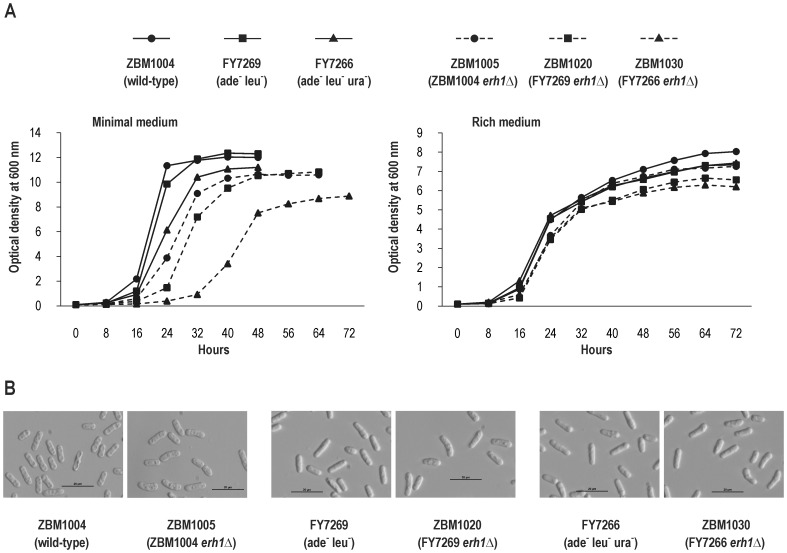
Effects of *erh1^+^* disruption in *S. pombe* on growth rate and cell morphology. (A) Growth curves for three *erh1^+^* strains (solid lines), prototrophic ZBM1004, auxotrophic (ade^−^ leu^−^) FY7269 and auxotrophic (ade^−^ leu^−^ ura^−^) FY7266 and three corresponding *erh1Δ* strains (dashed lines), ZBM1004 derivative ZBM1005, FY7269 derivative ZBM1020 and FY7266 derivative ZBM1030 in EMM2S minimal medium (left) or in YES rich medium (right). Cultures were inoculated to OD_600_ of 0.1 from stationary cultures in the same medium. (B) Cell morphology by light microscopy with Hoffman Modulation Contrast. Images of strains as in (A) cultured to stationary phase in YES.

When the strains were cultured in EMM2S minimal medium after transferring from the stationary phase all three disruptants displayed a delay in recovery from the stationary phase, grew more slowly and reached lower final optical densities (OD_600_) than did the corresponding *erh1^+^* strains (Figure 3A). The auxotrophic *erh1Δ* strains, especially ZBM1030 (ade^−^ leu^−^ ura^−^), exhibited a stronger growth defect than the prototrophic *erh1Δ* strain. When the strains were cultured in YES rich medium after transferring from the stationary phase the defect was only minimal for all three disruptants (Figure 3A).

The morphology of the *erh1Δ* disruptant cells was examined by light microscopy with Hoffman Modulation Contrast. Cells with a comma-like shape accumulated in cultures of all three disruptants when they reached the stationary phase in YES (Figure 3B) or EMM2S (data not shown).

**Figure 4:**
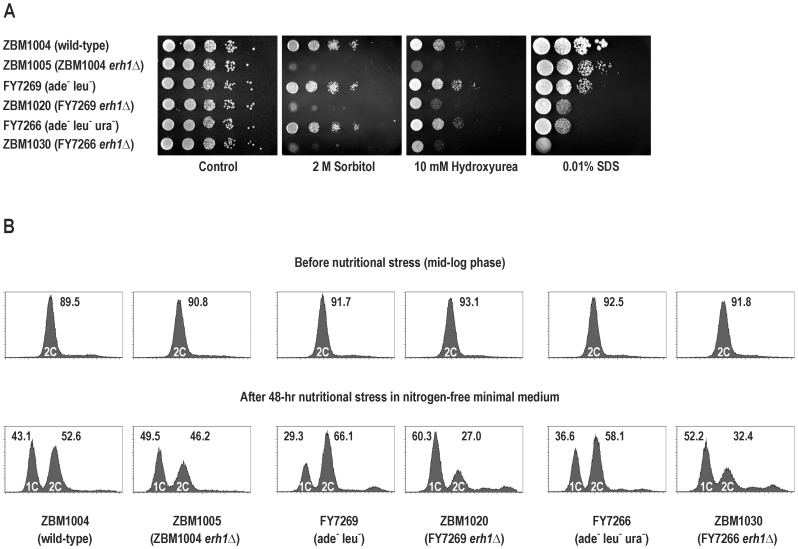
Effects of *erh1^+^* disruption on *S. pombe* sensitivity to stresses and on cell cycle progression. (A) Serial dilutions of cells were spotted on YES alone or YES supplemented with 2 M sorbitol, 10 mM hydroxyurea or 0.01% SDS agar plates and incubated for 5 days. Strains used: prototrophic *erh1^+^* ZBM1004 and its *erh1Δ* derivative ZBM1005, auxotrophic (ade^−^ leu^−^) *erh1^+^* FY7269 and its *erh1Δ* derivative ZBM1020, and auxotrophic (ade^−^ leu^−^ ura^−^) *erh1^+^* FY7266 and its *erh1Δ* derivative ZBM1030. (B) Cell cycle profiles following nutritional stress in nitrogen-free EMM2 minimal medium. Cells of strains as in (A) were stained with PI and sorted by flow cytometry. Upper series of profiles, cells before nutritional stress (in mid-log phase in YES); lower series of profiles, cells after 48-hour nutritional stress in EMM2-N. Peaks representing 1C and 2C DNA content are indicated. Numbers indicate the percentage of cells with a given DNA content.

To find other possible phenotypes associated with the *erh1Δ* mutation we studied the sensitivity of the *erh1Δ* strains to the following stress conditions: hyperosmotic stress (sorbitol), inhibition of DNA replication (hydroxyurea) and destabilization of the plasma membrane (SDS) using a serial dilution method (Figure 4A). Both auxotrophic *erh1Δ* strains showed hypersensitivity to all these agents. The prototrophic *erh1Δ* strain exhibited hypersensitivity to sorbitol similar to the auxotrophic *erh1Δ* strains. However, it was slightly less hypersensitive to hydroxyurea than the auxotrophic *erh1Δ* strains and showed only a minimal defect when grown in the presence of SDS.

We next analyzed the progression of the cell cycle in the *erh1Δ* strains using flow cytometry. The cell cycle of exponentially growing haploid fission yeast cells displays two unusual features. The G2 phase is very long, taking 70% of the generation time, and the completion of cytokinesis is delayed until the late S phase, which together results in approx. 90% of cells having a 2C DNA content (all G2, M and G1 phase cells) [24], [25]. However, following nitrogen deprivation, accumulation of uninucleate cells with a 1C DNA content is observed [24]. Switching from rich medium to nitrogen-free minimal medium leads to nutritional stress which is not only limited to nitrogen deprivation since the latter medium also lacks many organic compounds including amino acids and nucleotides. Thus, such transfer could also disturb the cell cycle. The *erh1Δ* strains cultured to the mid-log phase in YES exhibited 90.8–93.1% of cells with a 2C DNA content, the same as in the corresponding *erh1^+^* strains (Figure 4B). When subjected to nutritional stress in nitrogen-free EMM2 minimal medium for 48 hours, all strains showed a profile with an additional 1C peak. However, the proportion of cells with a 1C DNA content was lower in the auxotrophic *erh1^+^* strains than in the prototrophic *erh1^+^* strain (29.3% and 36.6% vs. 43.1% in FY7269, FY7266 and ZBM1004, respectively). Significantly, the auxotrophic *erh1Δ* strains exhibited a much higher percentage of 1C cells (52.2% and 60.3% in ZBM1030 and ZBM1020, respectively), showing that they were more readily arrested in the G1 phase in response to the nutritional stress than the corresponding *erh1^+^* strains. The prototrophic *erh1Δ* strain showed only a small increase (to 49.5%) in the proportion of 1C cells in comparison with the prototrophic *erh1^+^* strain.

### Complementation of the *erh1Δ* Mutation and Cross-species Experiments

**Figure 5:**
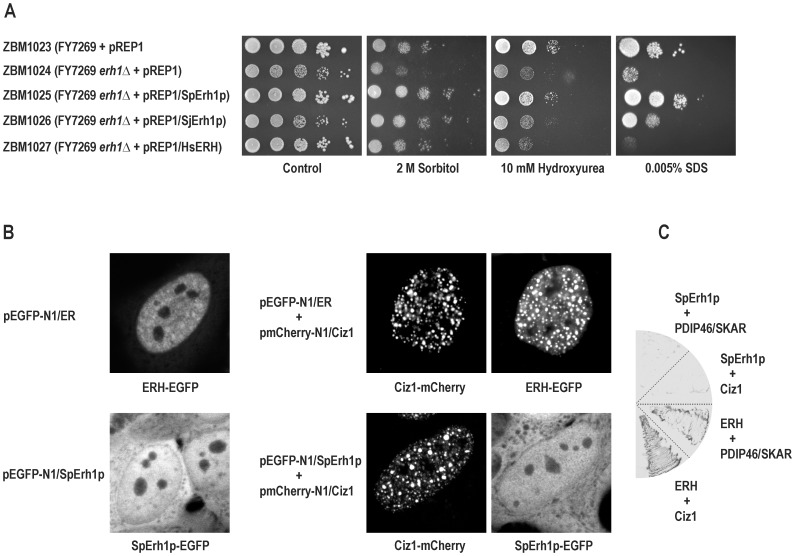
Complementation of *erh1Δ* mutation and cross-species experiments. (A) Serial dilutions of cells were spotted on EMM2+ADE alone or EMM2+ADE supplemented with 2 M sorbitol, 10 mM hydroxyurea or 0.005% SDS agar plates and incubated for 5 days. Strains used: auxotrophic (ade^−^ leu^−^) *erh1^+^* FY7269 transformed with pREP1 (ZBM1023), FY7269 *erh1Δ* derivative ZBM1020 transformed with pREP1 (ZBM1024), ZBM1020 transformed with pREP1/SpErh1p (ZBM1025), ZBM1020 transformed pREP1/SjErh1p (ZBM1026) and ZBM1020 transformed with pREP1/HsERH (ZBM1027). (B) Intracellular localization of SpErh1p in human HeLa cells by confocal microscopy. Upper series of images, cells transfected with plasmid coding for EGFP-tagged human ERH (pEGFP-N1/ER) alone or cotransfected with plasmid coding for mCherry-tagged human Ciz1 (pmCherry-N1/Ciz1); lower series of images, cells transfected with plasmid coding for EGFP-tagged SpErh1p (pEGFP-N1/SpErh1p) alone or cotransfected with pmCherry-N1/Ciz1. Direct fluorescence of EGFP or mCherry was observed in live cells. (C) Yeast two-hybrid analysis with SpErh1p used as bait. The host *S. cerevisiae* L40 cells coexpressing human ERH and Ciz1, human ERH and PDIP46/SKAR (both pairs as positive controls), SpErh1p and Ciz1, and SpErh1p and PDIP46/SKAR were lysed and the activity of the *lacZ* reporter gene (conversion of X-gal to a blue precipitate represented here as a strong gray color of lysed cells) was determined.

To exclude the possibility that the observed growth defects were not due to the *erh1Δ* disruption only, we tested the ability of an exogenous copy of *Sperh1^+^* to rescue these defects. We introduced plasmids pREP1 and pREP1/SpErh1p into strain ZBM1020 and the resulting transformants (strains ZBM1024 and ZBM1025, respectively) together with strain ZBM1023 were tested for growth in the presence of the same stress-inducing agents as above (Figure 5A). As expected, ZBM1024 exhibited hypersensitivity to each agent when compared to ZBM1023, and ZBM1025 showed no defect in the presence of hydroxyurea or SDS and even reduced sensitivity to sorbitol.

To find whether the functions are conserved despite the relatively low amino acid sequence identities between the ERH orthologs from the different species of the fission yeasts or between any fission yeast species and humans, we determined the ability of the *S. japonicus* and human *ERH* genes to complement the *erh1Δ* mutation in *S. pombe*. We introduced plasmids pREP1/SjErh1p and pREP1/HsERH into strain ZBM1020 and the resulting transformants (strains ZBM1026 and ZBM1027, respectively) were tested for growth on the same media as above (Figure 5A). While the *Sjerh1^+^* cDNA did rescue the *erh1Δ* growth defect in the presence of sorbitol or SDS, it was rather insufficient in the presence of hydroxyurea. The *ERH* cDNA rescued the *erh1Δ* mutant only in the presence of sorbitol.

To further study SpErh1p we performed heterologous expression of *Sperh1^+^*. Human HeLa cells were transfected with plasmids pEGFP-N1/SpErh1p and pEGFP-N1/ER coding for SpErh1p and human ERH, respectively, both tagged on their C-termini with EGFP, and were analyzed by fluorescence microscopy after 48 hours (Figure 5B). As expected, ERH localized exclusively to the nucleus [9], [11]. In contrast to the predominantly nuclear localization of SpErh1p in yeast cells (Figure 2B), this protein was equally present in the nucleus and cytoplasm of HeLa cells.

We next tested the ability of SpErh1p to interact with a universal molecular partner of human ERH, the Ciz1 protein which localizes to DNA replication foci in the nucleus. HeLa cells were cotransfected with plasmids pEGFP-N1/SpErh1p and pmCherry-N1/Ciz1 coding for human Ciz1 tagged on the C-terminus with the red fluorescent protein mCherry and analyzed by fluorescence microscopy (Figure 5B). HeLa cells cotransfected with pEGFP-N1/ER and pmCherry-N1/Ciz1 were used as a positive control. While ERH moved to the Ciz1-containing replication foci as reported previously [11], SpErh1p remained diffused in the nucleoplasm.

The possibility of interaction of SpErh1p with another universal molecular partner of human ERH, the PDIP46/SKAR protein, was tested using the yeast two-hybrid system which had been used to identify the both ERH interactors previously [9], [11]. The host *S. cerevisiae* strain L40 was transformed with plasmid pHybLex/Zeo-SpErh1p coding for SpErh1p fused to the C-terminus of the LexA DNA-binding domain. The LexA-SpErh1p chimeric protein alone did not activate the *LacZ* reporter gene in the bait strain (data not shown). Next, the bait strain was transformed with plasmid pYESTrp2/PDIP46 coding for human PDIP46/SKAR fused to the C-terminus of the B42 transcription activation domain. The bait strain was also transformed with plasmid pYESTrp2/Ciz1 coding for human Ciz1 fused to the C-terminus of B42. SpErh1p failed to interact with PDIP46/SKAR or Ciz1 (Figure 5C).

## Discussion

The *ERH* gene is present in genomes of most eukaryotic organisms including Animalia (from the sponge *Amphimedon queenslandica* to the tapeworm *Echinococcus multilocularis* to humans), Plantae (from the green alga *Chlorella* sp. to the moss *Physcomitrella patens* to the flowering plant *Arabidopsis thaliana*) and Protista (e.g., the protozoan *Toxoplasma gondii*, the red alga *Cyanidioschyzon merolae*, the water mold *Saprolegnia parasitica* or the slime mold *Dictyostelium discoideum*), which strongly suggests that it was present also in the genome of the last common ancestor of all eukaryotes (M.K. and P.K., unpublished data). In this respect, Fungi are striking exception since this gene has only been identified in the phyla Chytridiomycota and Zygomycota, often referred to as lower fungi. Here, we show that the *ERH* homolog is in fact present also in the genus *Schizosaccharomyces* that belongs to the phylum Ascomycota (Figure 1). However, the fission yeasts seem to be an exception among the higher fungi since, using the fission yeast *ERH* sequence, we were unable to identify an *ERH* homolog in the genome sequences of such fungi as *S. cerevisiae*, *A. nidulans* or *Neurospora crassa*, also belonging to Ascomycota, and the button mushroom *Agaricus bisporus*, the corn smut *Ustilago maydis* or the stem rust *Puccinia graminis*, all members of the phylum Basidiomycota (M.K. and P.K., unpublished data). The fission yeasts are a unique group of fungi as reflected by their placement into the separate class Schizosaccharomycetes which currently consists of only four species, *S. pombe*, *S. octosporus*, *S. cryophilus* and *S. japonicus* and diverged from the rest of Ascomycota very early in evolution [21], [26]–[28]. Thus, while the lower fungi and the fission yeasts still possess the *ERH* gene, it is plausible that most of the higher fungi have lost it during evolution. Conveniently, *S. pombe* is a fairly simple and extensively studied model organism, so the presence of *ERH* in its genome can greatly aid the elucidation of the ERH role in the eukaryotic cell, as was the case for numerous other genes and proteins.

We cloned the *ERH* genes from all four species of *Schizosaccharomyces*. The proteins coded by these genes show only 25–28% amino acid identity with human ERH, much less than the identity between human ERH and ERH from any other organism (Figure 1B). For example, human ERH displays 52% and 42% identities to the *Caenorhabditis elegans* and *A. thaliana* ERHs, respectively [3]. Furthermore, all four fission yeast ERHs show a unique four-amino acid deletion in the middle of the protein. Nevertheless, the secondary and tertiary structures of human ERH are preserved in Erh1p from *S. pombe* (Figure 1C). The fission yeast ERHs compared to each other display amino acid identities of between 85% (*S. cryophilus* vs. *S. octosporus*) and 52% (*S. cryophilus* vs. *S. japonicus*); the *S. pombe* ERH protein shows 72%, 71% and 57% identities to those from *S. octosporus*, *S. cryophilus* and *S. japonicus*, respectively (Figure 1B). Generally, the divergence of ERH sequences between species from a single genus is lower. In Vertebrata, the human, murine and *X. laevis* ERH proteins are identical; in the genus *Drosophila*, the ERH proteins of *D. melanogaster* and *D. virilis* display 95% identity [3], [4]. However, a recent analysis of all four fission yeast genomes has revealed that the average identities between all 1∶1 protein orthologs are only 55% for *S. pombe* and *S. japonicus* and 85% for *S. octosporus* and *S. cryophilus* [21]. The identities observed here between the fission yeast ERHs are in perfect agreement with these values and correctly reflect the phylogenetic relationships, with *S. cryophilus* and *S. octosporus* as the closest relatives, *S. pombe* as their nearest relative, and *S. japonicus* as the most divergent member of the genus [21], [23].

The fission yeast *ERH* genes have five (*Sperh1^+^*, *Soerh1^+^* and *Scerh1^+^*) or six (*Sjerh1^+^*) introns, which is significantly higher than in most of their other genes (Figure 1A). On average, there is approx. one intron per gene, with nearly half of them being intronless in all four species, and in *S. pombe* only approx. 3% of genes have five or more introns [7], [17], [21]. In contrast to the relatively low conservation of the amino acid sequence, the positions of introns are nearly perfectly preserved in the four *ERH* genes (Figure 1A). However, this phenomenon is not restricted to this gene only. Of all introns in the spliced 1∶1∶1∶1 orthologs, 81% share positions in all four species [21]. On the other hand, the fission yeast spliceosome seems to exhibit relatively low fidelity since during the cloning of *Sperh1^+^* and *Scerh1^+^* we detected incorrectly spliced transcripts (Figure 1D). Regarding the length, the incorrectly spliced introns do not differ from other fission yeast introns, which are within the range of 29–819 bp. Indeed, they are very close to the average intron length, 78 bp [7], [17]. Apparently, it is rather challenging for the spliceosomal machinery to remove as many as five introns from a pre-mRNA molecule of a gene with a very short ORF (in this case 312 bp). Notably, during the original annotation of the *S. pombe* genome the *ERH* gene was not identified but the authors predicted that some of the genes could remain undiscovered at that time if they have either a highly spliced structure with small exons or an ORF shorter than 100 aa [7].

As a first step toward characterization of the *S. pombe erh1^+^* gene we established its expression pattern in wild-type cells in a set of experimental conditions commonly studied in fission yeast. It turned out that this gene is expressed at a similar level in all conditions tested, regardless of the ploidy, growth stage, nutrient availability, and exposition to hyperosmotic stress (Figure 2A). In this respect, *erh1^+^* is similar to human *ERH* which is expressed ubiquitously [9]. To characterize the Erh1p protein we established its intracellular localization in fission yeast. The protein predominantly localized to the nucleus with the exception of the nucleolus, however, a cytoplasmic pool of the protein was seen as well (Figure 2B). This pattern resembles the intracellular localization of human ERH which, however, localizes almost exclusively to the nucleus (Figure 5B) [9]. We also demonstrated that Erh1p exists in two forms of different electrophoretic mobilities, both migrating more slowly than expected (Figure 2C). This probably reflects a posttranslational modification(s) of unknown nature.

With the aim of elucidating the ERH function in *S. pombe* we created strains with disruption of the *erh1^+^* gene in wild-type (prototrophic) and two auxotrophic (ade^−^ leu^−^ and ade^−^ leu^−^ ura^−^) genetic backgrounds. First, we looked for abnormalities during normal growth and noticed in the stationary phase in all three *erh1Δ* strains accumulation of cells with a comma-like shape, resembling the shape of mutants in *ban* genes defective in the maintenance of correct direction of cell growth (Figure 3B) [29]. Moreover, when transferred from the stationary phase to minimal medium (EMM2S) all three *erh1Δ* strains exhibited a delayed recovery and grew more slowly than did the corresponding *erh1^+^* strains (Figure 3A). That defect was the most profound in the auxotrophic backgrounds. The defect was also found in rich medium (YES), however, it was weak (Figure 3A).

When we tested the sensitivity of all three *erh1Δ* strains to sorbitol used to induce hyperosmotic stress, hydroxyurea used to inhibit DNA replication and SDS used to destabilize the plasma membrane and, in cells with a defective cell wall, to affect cell integrity, the *erh1Δ* disruptants in the auxotrophic backgrounds displayed strong hypersensitivity while the prototrophic *erh1Δ* strain showed a less pronounced defect (Figure 4A). Finally, in the auxotrophic *erh1Δ* disruptants an increased accumulation of uninucleate cells with a 1C DNA content was observed in response to nutritional stress in nitrogen-free EMM2 minimal medium (Figure 4B).

Thus, *erh1^+^* is not essential for vegetative growth in fission yeast, however, Erh1p may play a role in the cellular response to stress conditions. Since hypersensitivity to various stresses was observed it is likely that it could be part of a general stress response. In *S. pombe* this response seems to be controlled predominantly by a single regulatory system in which key roles are played by the stress-activated MAPK Sty1p (also known as Spc1p or Phh1p) and the transcription factor Atf1p [30], [31]. The *sty1^−^* mutants are hypersensitive to high osmolarity and hydroxyurea [32], [33]. However, in contrast to the *erh1Δ* mutant, the *sty1* ^−^ cells are more elongated than wild-type cells [34]. It appears that additional, less prominent systems are also involved in the stress response in fission yeast. Another MAPK, Pmk1p (also known as Spm1p), a constituent of the cell integrity pathway seems to be activated by various stress conditions as well [35]. The *pmk1Δ* mutant is hypersensitive to cell wall-damaging agents and displays delayed recovery from the stationary phase, however, it is not hypersensitive to high concentration of sorbitol despite Pmk1p being activated by this agent [35], [36]. Moreover, the *pmk1Δ* cells are shorter and more rounded [36]. The Tor1p kinase is also required for response to many stress conditions [37], [38]. The *tor1Δ* cells are hypersensitive to sorbitol [37]. In contrast to the *erh1Δ* mutants but similarly to the *sty1* ^−^ cells, the *tor1Δ* cells are more elongated [39]. Another TOR protein kinase, Tor2p, is necessary to pass through the G1 phase. However, the *tor2* ^−^ mutations promote arrest in G1 even in rich medium whereas the *erh1Δ* mutation facilitates arrest in G1 only on nutritional stress in nitrogen-free minimal medium [40]. In summary, a comparison of the *erh1Δ* phenotype with the effects of mutations in genes encoding key constituents of stress response has not indicated clearly in what pathway Erh1p could participate. On the other hand, the observed complex phenotype of the *erh1Δ* mutation could be simply viewed as a general diminishing of the fitness of the cells.

During normal growth, the phenotype of the *erh1Δ* mutation was manifested in both prototrophic and auxotrophic backgrounds but when yeast cells were exposed to stresses, the effects were mainly seen in the auxotrophic backgrounds. Kawai *et al*. suggested that some growth defects of the *tor1Δ* mutation could result from an inability to uptake nutrients efficiently [37]. Others showed that growth of leucine auxotrophs, uracil auxotrophs and, to a lesser extent, histidine auxotrophs but not adenine auxothrophs on minimal medium, that is, in the presence of a high concentration of ammonia, was inhibited by rapamycin, a drug inhibiting TOR kinases, and that the rapamycin-treated *tor1^+^* cells or *tor1Δ* cells were defective in leucine uptake, which correlated with reduced expression of three amino acid permeases [41]. We do not think that we observed exactly the same phenomenon with the *erh1Δ* mutation in the auxotrophic backgrounds for at least two reasons. Firstly, the *erh1Δ* growth defects were also displayed in rich medium which does not contain a high concentration of ammonia (Figure 4A). Secondly, although these defects were also observed in minimal medium in the *ade6-M210 leu1-32* background (Figure 5A), adenine was the only supplement added, since the leucine auxotrophy was complemented with the *LEU2* gene on the pREP1 plasmid. Interestingly, Tor2p is also involved in regulation of expression of genes coding for amino acid and purine permeases and transporters. In contrast to the *tor1Δ* cells, in the *tor2* ^−^ cells numerous such genes were induced [40]. One explanation of the defects observed mainly in the auxotrophic backgrounds could be that supplementation with an auxotrophy-complementing amino acid or nucleobase even at a high concentration does not completely alleviate the auxotrophic defect which, combined with the *erh1Δ* mutation, may result in a synthetic growth defect in the presence of some agents. The *ERH* gene was originally identified in *D. melanogaster* as a mutation that was manifested only in the *r* mutant background [1]. The *r* gene codes for the first three enzymatic activities of the *de novo* pyrimidine nucleotide biosynthetic pathway and its mutation resulted in pyrimidine deficiency [10]. Our results show that in fact, the effects of the *erh1Δ* mutation are fully manifested only in auxotrophic backgrounds, however, both auxotrophic *erh1Δ* strains (ade^−^ leu^−^ and ade^−^ leu^−^ ura^−^) behaved in the very similar manner. Thus, in *S. pombe*, pyrimidine auxotrophy is not required for the development of the full *erh1Δ* phenotype.

The complementation of the *erh1Δ* mutation with an exogenous copy of *Sperh1^+^* showed that the observed effects could be attributed solely to that mutation (Figure 5A). The proteins encoded by the *Sjerh1^+^* and human *ERH* genes display only 57% and 28% identities with SpErh1p, respectively (Figure 1B). These genes complemented the *erh1Δ* mutation in a manner that reflected their similarity to *Sperh1^+^* (Figure 5A). The closer homolog rescued the *erh1Δ* mutant both in the presence of sorbitol and SDS, while the more distant one only complemented *erh1Δ* in the presence of sorbitol. Neither homolog could rescue the *erh1Δ* defect in the presence of hydroxyurea. This finding may reflect the interactions of SpErh1p with different partners in response to distinct stresses and, depending on their similarity to SpErh1p, the heterologous ERH proteins could interact with only some of them. No interactor of SpErh1p has been identified yet, however, there are no obvious homologs of the known universal interactors of human ERH, Ciz1 and PDIP46/SKAR, in the fission yeast proteome (M.K. and P.K., unpublished data).

Due to the moderate degree of identity of SpErh1p with human ERH, yet almost identical secondary and tertiary structure (Figure 1B, C), its localization to the nucleus (Figure 2B) and the fact that *ERH* complemented at least partially the *erh1Δ* mutation (Figure 5A), SpErh1p could be a valuable molecular tool to study the interaction of ERH with its two nuclear partners, Ciz1 and PDIP46/SKAR, e.g., in searching for amino acid residues responsible for the interactions. We found that SpErh1p expressed in HeLa cells did enter the nucleus (Figure 5B), but in contrast to ERH, it was not recruited to the Ciz1-containing replication foci suggesting a lack of an interaction between SpErh1p and Ciz1. No interaction between SpErh1p and PDIP46/SKAR and also between SpErh1p and Ciz1 was found by yeast two-hybrid system (Figure 5C). These results suggest that the amino acid residues shared between ERH and SpErh1p are not sufficient for an interaction with Ciz1 or PDIP46/SKAR and further studies are necessary to identify the ERH residues responsible for these interactions.

In conclusion, we posit that the ERH protein was present in the nucleus of the last common ancestor of all eukaryotes in which it played a role in the response to adverse environmental conditions. However, the *ERH* gene was not essential for normal growth and during evolution of eukaryotes, ancestors of some species, including most of the higher fungi have lost this gene, possibly because its role could be fulfilled by another protein. In the higher fungi several independent *ERH* loss events must have occurred, since the divergence of Ascomycota and Basidiomycota obviously preceded the separation of the class Schizosaccharomycetes from the rest of Ascomycota. On the other hand, with an increase of the proteome size and accumulation of changes in the primary structure of ERH, the protein became involved in interactions with some novel acquisitions, i.e., Ciz1 and PDIP46/SKAR in Vertebrata and gained additional functions while the primary one could be restricted or even completely eliminated in some evolutionary lineages. In turn, the complexity of its interactions constrained further evolution of this small, single-domain protein.

## Materials and Methods

### Databases and Bioinformatic Analyses

For *S. pombe* the genome sequence at the Sanger Institute (www.sanger.ac.uk/Projects/S_pombe) was used. For *S. octosporus*, *S. cryophilus* and *S. japonicus* the collection of fission yeast genome sequences available at the Broad Institute (www.broadinstitute.org/annotation/genome/schizosaccharomyces_group) was employed. DNA analyses were performed with the BLAST program [42]. Amino acid sequence alignments were performed using the Clustal X 2.0 program and manual adjustment [43]. Protein structures were predicted by the SWISS-MODEL server (swissmodel.expasy.org) using the Protein Data Bank (www.rcsb.org/pdb) coordinates for the human ERH protein (PDB identifier: 2nmlA) [44]. Protein images were produced with the UCSF Chimera package [45].

### General Techniques for the Fission Yeasts

The fission yeast strains used in this study are listed in Table 1. Standard methods for *S. pombe* were used as described [18]. All four species were grown on YES (YE rich medium supplemented with adenine, uracil, leucine, lysine and histidine, each at 225 mg/l) at 30°C, with the exception of *S. cryophilus* which was cultivated at 25°C. For *S. pombe* the following variations of the EMM2 minimal medium were also used: EMM2S (EMM2 supplemented as above), EMM2+ADE (EMM2 supplemented with adenine at 100 mg/l), EMM2-N (nitrogen-free EMM2) and low-glucose EMM2 (EMM2 with a glucose concentration lowered to 0.5%). For growth curve determination two-and-a-half-day YES or EMM2S precultures were diluted to a starting OD_600_ of 0.1 in YES and EMM2S, respectively, and grown with shaking for up to 72 hours (until the stationary phase was reached). For growth tests cells were cultured to the mid-log phase in YES or EMM2+ADE, washed with saline and ten-fold serial dilutions were spotted on plates with YES or EMM2+ADE agar alone and YES or EMM2+ADE agar supplemented with 2 M sorbitol, 10 mM hydroxyurea or 0.005–0.01% SDS and were grown for 5 days. For nutritional stresses cells were cultured to the mid-log phase in YES, washed three times with saline and transferred to EMM2-N and incubated with shaking for 48 hours or transferred to low-glucose EMM2 and incubated with shaking for 24 hours. For hyperosmotic stress cells were cultured to the mid-log phase in YES, transferred to YES supplemented with 2 M sorbitol and incubated with shaking for 24 hours. Transformations of strains FY7269 and ZBM1020 with pREP1 or derivatives were done by the lithium acetate method with sonicated salmon sperm DNA as carrier and incubation with DMSO, and transformants were selected on EMM2+ADE agar. For construction of a diploid, strains FY12697 and FY7519 were crossed on ME agar at 25°C and after streaking out on YES agar supplemented with Phloxin B (5 µg/ml) diploid clones (stained dark red) were identified. For construction of the prototrophic haploid ZBM1004, strains FY7269 and FY7519 were crossed as above and the ade^+^ leu^+^ meiotic progeny was selected on EMM2 agar followed by determination of the mating type by polymerase chain reaction (PCR) with the common (5′-AGAAGAGAGAGTAGTTGAAG-3′), *h^+^* specific (5′-ACGGTAGTCATCGGTCTTCC-3′) and *h* ^−^ specific (5′-TACGTTCAGTAGACGTAGTG-3′) primers.

**Table 1:** Fission yeast strains used in this study.

Name	Genotype/description	Source
	*S. japonicus*	
NIG2021	a single colony isolate from type strain IFO 1609	NIG^1^ [51]
	*S. octosporus*	
DSMZ70573	Windisch isolate	DSMZ^2^
	*S. cryophilus*	
CBS11777	type strain	CBS^3^ [23]
	*S. pombe*	
FY7266	*h* ^−^ *ade6-M210 leu1-32 ura4-D18*	YGRC^4^
FY7269	*h* ^−^ *ade6-M210 leu1-32*	YGRC
FY7519	*h* ^+^ (Leupold standard strain L975)	YGRC
FY12697	*h* ^−^ (Leupold standard strain L972)	YGRC
ZBM1004	*h* ^−^	This study
ZBM1005	*h* ^−^ *erh1*::*kanMX6*	This study
ZBM1020	*h* ^−^ *ade6-M210 leu1-32 erh1*::*kanMX6*	This study
ZBM1021	*h* ^−^ *ade6-M210 leu1-32* [pREP1/SpErh1p-yEGFP]	This study
ZBM1022	*h* ^−^ *ade6-M210 leu1-32* [pREP1/SpErh1p-3HA]	This study
ZBM1023	*h* ^−^ *ade6-M210 leu1-32* [pREP1]	This study
ZBM1024	*h* ^−^ *ade6-M210 leu1-32 erh1*::*kanMX6* [pREP1]	This study
ZBM1025	*h* ^−^ *ade6-M210 leu1-32 erh1*::*kanMX6* [pREP1/SpErh1p]	This study
ZBM1026	*h* ^−^ *ade6-M210 leu1-32 erh1*::*kanMX6* [pREP1/SjErh1p]	This study
ZBM1027	*h* ^−^ *ade6-M210 leu1-32 erh1*::*kanMX6* [pREP1/HsERH]	This study
ZBM1028	*h* ^−^ *ade6-M210 leu1-32 erh1-yEGFP*::*kanMX6*	This study
ZBM1030	*h* ^−^ *ade6-M210 leu1-32 ura4-D18 erh1*::*kanMX6*	This study

1 The National Institute of Genetics, Japan.

2 Deutsche Sammlung von Mikroorganismen und Zellkulturen, Germany.

3 Centraal Bureau voor Schimmelcultures, the Netherlands.

4 The Yeast Genetics Resource Center, Japan.

### Total DNA, RNA and cDNA

Total DNA was liberated from cells by heating their suspension in water for 5 minutes at 95°C and removing cellular debris by spinning at 13200×*g* for 3 minutes at room temperature. For total RNA preparation, cells were pulverized in liquid nitrogen and RNA was isolated using TRI Reagent (Sigma) followed by purification with NucleoSpin RNA Clean-up (Macherey-Nagel). For total cDNA preparation, 2 µg of total RNA was used with RevertAid M-MuLV Reverse Transcriptase (Fermentas) according to the manufacturer’s recommended protocol.

### Northern Analysis

Total RNA was resolved on a 1.2% formaldehyde/agarose gel using RiboRuler High Range RNA Ladder (Fermentas) as RNA size standards and transferred to Hybond-N+ nylon membrane (Amersham) by capillary blotting. The cloned *Sperh1^+^* cDNA was labeled with [α-^32^P]dATP (Hartmann Analytic) using Megaprime DNA labelling system (Amersham) and high stringency hybridization was carried out overnight at 65°C in the modified Church and Gilbert buffer according to the protocol recommended by Amersham. After washing, the membrane was exposed for one week to an X-ray film with an intensifying screen at −80°C.

### Immunoprecipitation

Yeast cells in the early stationary phase from 2 liters of EMM2+ADE were pelleted, frozen in liquid nitrogen and pulverized manually. The obtained powder was suspended in lysis buffer (30 mM Tris-HCl, 150 mM NaCl, 0.05% NP-40, 1 mM PMSF, pH 8.0), the suspension was cleared by centrifugation (40000×*g* for 60 minutes at 4°C) and the supernatant was dialyzed for 3 hours against the same buffer supplemented with 10% glycerol. Lysates were incubated with anti-HA epitope monoclonal antibody (F-7, Santa Cruz) followed by incubation with protein A-coated beads (Santa Cruz). After washing four times with the lysis buffer, immunoprecipitates were resolved on a 15% SDS/polyacrylamide gel and stained with silver as described previously [9].

### Cloning of the *ERH* Genes and Plasmid Constructs

All amplifications by PCR were performed with a high-fidelity *PfuTurbo* hotstart DNA polymerase (Stratagene) and all constructs were verified by automated DNA sequencing. Cloning was performed according to standard procedures [46]. All genomic copies and cDNAs of the fission yeast *ERH* genes were amplified employing as templates total DNA or total cDNA isolated from wild-type strains (the first four strains in Table 1) and the following pairs of primers: forward (5′-GCGGAATTCCCTTGCTCGATAAAAACAG-3′) and reverse (5′-GCGCTGCAGATTTAAAGCATAGCAATGAG-3′) for *S. pombe*, forward (5′-CGCGAATTCTTCGTTCCATCATTCCTC-3′) and reverse (5′-GCGCTGCAGAATAATAGTATTTCAACGAA-3′) for *S. octosporus*, forward (5′-GCGGAATTCCCTTTCCATTGCTCCTCTGTC-3′) and reverse (5′-GCGCTGCAGCAGTAGGCGACATTAGAAGAT-3′) for *S. cryophilus* and forward (5′-GCGGAATTCCGTGAAAACACGTACGCG-3′) and reverse (5′-GCGCTGCAGTGAGACATACAAGAAAATAAG-3′) for *S. japonicus*; restriction sites introduced for cloning are underlined. Amplified DNA fragments were ligated into pUC18 (Amersham) at the EcoRI and PstI sites yielding plasmids with *ERH* genomic copies (pUC18/Sperh1, pUC18/Soerh1, pUC18/Scerh1 and pUC18/Sjerh1; GenBank accession numbers: JN194210, JN194212, JN194213 and JN194211, respectively) and plasmids with *ERH* cDNAs (pUC18/cSperh1, pUC18/cSoerh1, pUC18/cScerh1 and pUC18/cSjerh1; GenBank accession numbers: JN194206, JN194208, JN194209 and JN194207, respectively).

pFA6a-5′arm(Sperh1)-Kan-3′arm(Sperh1). The region of 271 bp immediately downstream from the stop codon of *Sperh1^+^* was amplified from total DNA from *S. pombe* with forward (5′-GCGAATTCAAATATCGAACACCATATTATG-3′) and reverse (5′-GCACTAGTAGACAAATGCCATTTATTAGAAC-3′) primers and ligated into pFA6a-link-yEGFP-Kan [47] at the EcoRI and BcuI sites yielding pFA6a-link-yEGFP-Kan-3′arm(Sperh1). Similarly, the region of 201 bp immediately upstream from the start codon of *Sperh1^+^* was amplified with forward (5′-GCGTCGACTCGCTACTCAATAACTGTGCTAC-3′) and reverse (5′-GCAGATCTTACGAGCCAAGTATTTTGAAG-3′) primers and used to replace the sequence coding for link-yEGFP in pFA6a-link-yEGFP-Kan-3′arm(Sperh1) by employing SalI and BglII.

pFA6a-3′ORF(Sperh1)-link-yEGFP-Kan-3′arm(Sperh1). The region of 219 bp immediately upstream from the stop codon of *Sperh1^+^* was amplified from total DNA from *S. pombe* with forward (5′-GCGCTCGAGGAATCTATTGATGTCTCAA-3′) and reverse (5′-GCGCTCGAGAAGGGATCTGTCTAGCAGC-3′) primers and ligated into pFA6a-link-yEGFP-Kan-3′arm(Sperh1) at the SalI site.

pREP1/SpErh1p-link-yEGFP. The *Sperh1^+^* ORF was amplified from pUC18/cSperh1 with forward (5′-GATGTCGACCGTAATGTCGCCTCCACCAGC-3′) and reverse (5′-GATCCCGGGAGGGATCTGTCTAGCAG-3′) primers and ligated into pREP1 [48] at the SalI and Cfr9I sites yielding pREP1/SpErh1-nsc. The sequence coding for link-yEGFP was amplified from pFA6a-link-yEGFP-Kan with forward (5′-GCGCCCGGGGTGATCGGTGACGGTGCTGGTTTA-3′) and reverse (5′-GCGCCCGGGTTATTTGTACAATTCATCCAT-3′) primers and ligated into pREP1/SpErh1-nsc at the Cfr9I site.

pREP1/SpErh1p-3HA. The sequence coding for 3HA was amplified from pFA6a-3HA-His3MX6 [49] with forward (5′-CAGCCCGGGAACATCTTTTACCCATAC-3′) and reverse (5′-GAGCCCGGGTCAGCACTGAGCAGCG-3′) primers and ligated into pREP1/SpErh1-nsc at the Cfr9I site.

pREP1/SpErh1p. The *Sperh1^+^* CDS was amplified from pUC18/cSperh1 with forward (5′-GATGTCGACCGTAATGTCGCCTCCACCAGC-3′) and reverse (5′-GATCCCGGGTTAAGGGATCTGTCTAGC-3′) primers and ligated into pREP1 at the SalI and Cfr9I sites.

pREP1/SjErh1p. The *Sjerh1^+^* CDS was amplified from pUC18/cSjerh1 with forward (5′-GCGGTCGACTATGTCATTTGCATCCATGG-3′) and reverse (5′-GATCCCGGGTTATGCAGATACTTCCTCAT-3′) primers and ligated into pREP1 at the SalI and Cfr9I sites.

pREP1/HsERH. The human *ERH* CDS was amplified from pCR2.1/ER [9] with forward (5′-GATGTCGACCGTAATGTCTCACACCATT-3′) and reverse (5′-GATCCCGGGTTATTTCCCAGCCTG-3′) primers and ligated into pREP1 at the SalI and Cfr9I sites.

pEGFP-N1/SpErh1p. The *Sperh1^+^* ORF was amplified from pUC18/cSperh1 with forward (5′- GAGCTCGAGCACGATGTCGCCTCCACCAGC-3′) and reverse (5′- GCGCTGCAGAGGGATCTGTCTAGCAGCTG-3′) primers and ligated into pEGFP-N1 (Clontech) at the XhoI and PstI sites.

pHybLex/Zeo-SpErh1p. The *Sperh1^+^* CDS was amplified from pUC18/cSperh1 with forward (5′-GCGGAATTCTCGCCTCCACCAGCAGAATC-3′) and reverse (5′-GCGCTCGAGTTAAGGGATCTGTCTAGCAGC-3′) primers and ligated into pHybLex/Zeo (Invitrogen) at the EcoRI and XhoI sites.

pEGFP-N1/ER, pmCherry-N1/Ciz1, pHybLex/Zeo-ER, pYESTrp2/Ciz1 and pYESTrp2/PDIP46 were described previously [9], [11].

### Disruption of the *Sperh1^+^* Gene

A full-length MX6-type disruption cassette of 1924 bp was amplified by PCR from pFA6a-5′arm(Sperh1)-Kan-3′arm (Sperh1) with forward (5′-TCGCTACTCAATAACTGTGCTAC-3′) and reverse (5′-AGACAAATGCCATTTATTAGAAC-3′) primers and introduced into *S. pombe* cells (strains ZBM1004, FY7269 and FY7266) by chemical transformation. After overnight incubation in YES, transformants were selected on YES agar supplemented with Geneticin (G418 sulfate, Gibco BRL, 150 µg/ml) and verified by PCR using total DNA and forward (5′-TAAATTGTGAGAACTGCAAAG-3′) and reverse (5′-GGAATTGGTCCTTTGTCTTGC-3′) primers that anneal to the genomic DNA outside of the 5′ and 3′ arms (for the presence of the 2074-bp PCR product).

### Epitope Tagging of the *Sperh1^+^* Gene in its Chromosomal Locus

*Sperh1* was chromosomally tagged with yEGFP in strain FY7269 by employing pFA6a-3′ORF(Sperh1)-link-yEGFP-Kan-3′arm (Sperh1) as a template, forward (5′-AGGAATCTATTGATGTCTCAA-3′) and reverse (5′-AGACAAATGCCATTTATTAGAAC-3′) primers and the same strategy as used for the gene disruption.

### Microscopy

Images of yeast cells were acquired on a Nikon Eclipse TE200 microscope with Hoffman Modulation Contrast, HMC LWD 40×/0.55 objective and DS-Fi1 CCD camera head or a Nikon Eclipse Ti-E fluorescence microscope with Nomarski Interference Contrast, Apo TIRF 100×/1.49 Oil DIC objective and DS-5Mc CCD camera head. For staining with DAPI, 2.5 µl of yeast cell suspension in water was spread on a microscope slide, fixed on a heating plate at 65°C for a few seconds (until water just evaporated) and 2.5 µl mounting medium with DAPI (UltraCruz, Santa Cruz) was added followed by covering with a 16 mm square coverslip. Images of HeLa cells expressing fluorescent proteins were acquired on a Zeiss LSM510 confocal microscope as described previously [9], [11].

### Flow Cytometry

Yeast cells were stained with propidium iodide (PI) according to [25], [50]. Briefly, after fixing in 70% ethanol cells were washed with 50 mM sodium citrate, pH 7.0, suspended in the same buffer supplemented with ribonuclease A (100 µg/ml) and incubated for 2 hours at 37°C followed by adding an equal volume of the same buffer supplemented with PI (final concentration of 4 µg/ml) and short sonication bursts. Cells were sorted based on DNA content using a Becton-Dickinson FACSCalibur flow cytometer and cell cycle analysis was performed with the CellQuest Pro software.

### Other Methods

Maintenance and transfection of HeLa cells (ECACC 93021013), yeast two-hybrid analysis and protein identification by tandem mass spectrometry were performed as described previously [9], [11].
